# MRI for the assessment of malignancy in BI-RADS 4 mammographic microcalcifications

**DOI:** 10.1371/journal.pone.0188679

**Published:** 2017-11-30

**Authors:** Barbara Bennani-Baiti, Matthias Dietzel, Pascal A. Baltzer

**Affiliations:** 1 Department of Pharmaceutical Chemistry, University of Vienna, Vienna, Austria; 2 Department of Biomedical Imaging and Image-guided Therapy, Vienna General Hospital (AKH), Medical University of Vienna, Vienna, Austria; 3 Department of Radiology, University of Erlangen-Nürnberg, Nürnberg, Germany; University of South Alabama Mitchell Cancer Institute, UNITED STATES

## Abstract

**Purpose:**

Assess the performance of breast MRI to diagnose breast cancer in BI-RADS 4 microcalcifications detected by mammography.

**Materials and methods:**

This retrospective, IRB-approved study included 248 consecutive contrast-enhanced breast MRI (1.5T, protocol in accordance with EUSOBI recommendations) performed to further diagnose BI-RADS 4 microcalcifications detected at mammography during a 3-year period. Standard of reference had to be established by histopathology. Routine consensus reading results by two radiologists were dichotomized as positive or negative and compared with the reference standard (benign vs malignant) to calculate diagnostic parameters.

**Results:**

There were 107 malignant and 141 benign microcalcifications. Malignancy rates were 18.3% (23/126 BI-RADS 4a), 41.7% (25/60 BI-RADS 4b) and 95% (59/62 BI-RADS 4c). There were 103 true-positive, 116 true-negative, 25 false-positive, and 4 false-negative (one invasive cancer, three DCIS; 2 BI-RADS 4c, 1 BI-RADS 4b on mammography) breast MRI findings, effecting a sensitivity, specificity, PPV, and NPV of 96.3% (95%-CI 90.7–99.0%), 82.3% (95%-CI 75.0–88.2%), 80.5% (95%-CI 72.5–87.0%) and 96.7% (95%-CI 91.7–99.1%), respectively.

**Conclusion:**

MRI is an accurate tool to further diagnose BI-RADS 4a and 4b microcalcifications and may be helpful to avoid unnecessary biopsies in BI-RADS 4a and 4b lesions. BI-RADS 4c microcalcifications should be biopsied irrespective of MRI findings.

## Introduction

Mammographic microcalcifications are detected in about one third of screening mammograms and are found in up to 40% of breast cancers [1, 2]. Since approximately 80% of ductal carcinoma in situ (DCIS) present only as microcalcifications at mammography, mammographic microcalcifications represent an important imaging hallmark that need to be further evaluated [1, 3].These lesions are currently further assessed mostly by percutaneous biopsy or by follow-up. Mammographic microcalcifications are rated according to Breast Imaging Reporting and Data System (BI-RADS) criteria. The most recent BI-RADS lexicon has eliminated BI-RADS 3 rating for such lesions. This is due to the wide range of malignancy rates that was found for BI-RADS 3 mammographic microcalcifications (0–9.7%) that do not comply with the BI-RADS definition of BI-RADS 3 lesions, which are characterized with a disease prevalence of less than 2% [4, 5]. Reported disease prevalence for BI-RADS 4 mammographic microcalcifications range from 32 to 65.2% and from 91.4 to 100% for BI-RADS 5 rated lesions [5]. Reported Positive Predictive Values (PPVs) for BI-RADS 4 lesions are low, ranging from 20–65.2% as assessed by histopathology [6–9]. They display great heterogeneity, probably owing to data being pooled from different subgroups (BI-RADS 4a-4c). Since, there is no safe established additional screening tool to further differentiate these lesions and since malignancy rates are overall too high to justify follow-up alone, almost all mammographic microcalcifications undergo biopsy to exclude malignancy. Consequently, to ensure the safety of the cohort, about 34.8–62% of patients with BI-RADS 4 rated mammographic microcalcifications undergo biopsy for a benign result [5]. Since Magnetic Resonance Imaging (MRI) has been shown to exhibit the highest sensitivity and specificity for breast cancer detection in general and an Negative Predictive Value (NPV) of nearly 100% in non-calcified lesions referred for problem solving [10], several studies have tried to see whether MRI can further evaluate mammographic microcalcifications. A recent meta-analysis has analysed their findings and concluded that MRI may indeed be helpful in the assessment of BI-RADS 4 microcalcifications pending on the underlying disease prevalence [5]. As indicated above, reported BI-RADS 4 lesion disease prevalence varies and increases from BI-RADS 4a to BI-RADS4c lesions. Therefore, we sought to investigate whether MRI can safely exclude malignancy in BI-RADS 4 lesions according to the respective BI-RADS 4 rating (a-c).

## Materials and methods

### Patient selection

For this cross-sectional single-center Institutional Review Board approved retrospective study, 2472 patients undergoing breast MRI during a period of 36 months at the university hospital Jena were eligible. To identify patients with mammographic microcalcifications, we queried our institutional database. This database included the date of examination, if present, the lead finding (i.e. mass, microcalcification, asymmetric density, architectural distortion), and the assigned BI-RADS category. All patients that were referred for breast MRI evaluation of mammographic microcalcifications rated BI-RADS 4a-4c at full field digital mammography followed by subsequent histopathological diagnosis were selected. Staging examinations of biopsy-proven cancers were excluded. In case of benign findings, a follow-up of at least 24 months had to be documented. Consequently, 248 consecutive patients met our inclusion criteria.

### MR imaging

MR imaging was performed in prone position on 1.5 Tesla units (Magnetom Avanto and Magnetom Sonata, Siemens Medical Solutions, Erlangen, Germany) with dedicated vendor-supplied four-channel bilateral breast coils (Siemens Medical Solutions, Erlangen, Germany). The MRI-protocol was designed following international recommendations [11, 12] and employed a dynamic sequence (T1-weighted FLASH 2D, GRAPPA factor 2, repetition time 113 ms, echo time 5 ms, flip angle 80°, spatial resolution 1.1 x 0.9 x 3 mm, 33 slices) with 1-minute temporal resolution performed once before and seven times after automated intravenous injection (3 ml/s, Spectris, Medrad, Pittsburgh, USA) of 0.1 mmol/kg Gd-DTPA (Magnevist, Bayer Health Care, Leverkusen, Germany). Subtractions were calculated by subtracting precontrast from postcontrast sequences. The protocol was completed by a bilateral axial T2-weighted Turbo-Spin-Echo sequence (T2w-TSE, GRAPPA factor 2, TR 8900 ms, TE 207 ms, flip angle 90°, spatial resolution 0.8 x 0.7 x 3 mm, 33 slices, time of acquisition 2:15 minutes), a bilateral axial Turbo Spin Echo Inversion Recovery sequence with magnitude reconstruction (TIRM, GRAPPA factor 2, TR 8420 ms, TE 70 ms, TI 150 ms, flip angle 180°, spatial resolution 1.7 x 1.4 x 3 mm, 33 slices, time of acquisition 2:33 minutes) acquired in identical slice positions. Additionally, Diffusion Weighted Imaging (DWI) (Echo Planar Imaging, EPI, GRAPPA factor 2, TR 3500, TE 80, echo distance 0.95 ms, 6 averages, 3 b-values: 0, 750, 1000 s/mm2, spectral fat saturation, spatial resolution 1.8 x 1.8 x 6 mm, 22 slices, time of acquisition 2:38 minutes) was carried out.

### Reference standard

Histopathological diagnosis was obtained after percutaneous image-guided biopsy followed by subsequent surgery in case of malignant diagnosis. Malignant lesions were further subcategorized into ductal carcinoma in situ (DCIS), invasive carcinomas with extensive intraductal component (EIC) and invasive carcinomas (IC). Benign lesions as identified by histopathological result were either operated on (upon either patient or surgeon’s request) and/or followed-up by imaging (at least mammography and ultrasound) for a minimum of 24 months. Board-certified breast pathologists with extensive experience in the field determined histopathological results. In accordance with national S3-guidelines, specimen were tested for hormonal receptors (Human epidermal growth factor receptor 2 (HER2/neu), progesterone receptor (PR), and estrogen receptor (ER)).

### Data analysis

All MR-images were chronologically analyzed by a consensus double-reading approach of two experienced radiologists (> 500 Breast MRI examinations/year) with access to mammography results during routine clinical practice. Vacuum-assisted biopsy was always performed after breast MRI to avoid artifacts, thus MRI reading was done before histopathological diagnosis. All examinations were assigned a consensus MRI BI-RADS category. MRI BI-RADS findings were dichotomized into positive (BI-RADS 4–5) and negative (BI-RADS 1–3) results and ensuing true negative (TN), false negative (FN), true positive (TP) and false positive (FP) numbers were taken from cross-tabulations against the final diagnosis for further statistical analysis. The main lesion (one per patient) was analyzed for the purpose of this study.

### Statistical analysis

Diagnostic parameters were calculated using MedCalc^®^ and SPSS 22.0 (IBM, USA) [13]. Sensitivity, Specificity, PPV and NPV were determined along with their respective 95%-confidence intervals. Fisher’s exact test was used to probe for differences between BI-RADS 4a-c subgroups. P-values below 0.01 were deemed to characterize significant findings for all calculations.

## Results

### Patient and lesion characteristics

248 patients, accounting for 141 benign and 107 malignant lesions were included in this retrospective IRB approved study. This equaled a disease prevalence of 43.1%. 33.9% of the investigated lesions represented invasive malignant lesions. Mean patient age was 60 years (+/- 11 years; range 31–82 years). Patients were referred to MRI for the assessment of BI-RADS 4a-c mammographic microcalcifications.

Malignant lesion distribution, grading and receptor status are given in Table 1 and Table 2. All underlying data are given in the S1 File.

**Table 1:** Immunohistochemical profile of malignant lesions.

		IC	EIC	DCIS	total
**ER**	positive	10 (33.3%)	10 (33.3%)	10 (33.3%)	30 (100%)
	negative	39 (50.6%)	25 (32.5%)	13 (16.9%)	77 (100%)
**PR**	positive	36 (48.0%)	28 (37.3%)	11 (14.7%)	75 (100%)
	negative	13 (40.6%)	7 (21.9%)	12 (37.5%)	32 (100%)
**Her2/neu**	positive	15 (41.7%)	10 (27.8%)	11 (30.6%)	36 (100%)
	negative	34 (47.9%)	25 (35.2%)	12 (16.9%)	71 (100%)

ER: Estrogen Receptor; PR: Progesterone Receptor; Her2/neu: human epidermal growth factor receptor 2; IC: Invasive Cancer; EIC: Extensive Intraductal Component; DCIS: Ductal Carcinoma In Situ

**Table 2:** Malignant lesion grading distribution.

	IC	EIC	DCIS	total
G1	2 (33.0%)	2 (33.0%)	2 (33.0%)	6 (100%)
G2	19 (47.5%)	12 (30.0%)	9 (22.5%)	40 (100%)
G3	28 (45.9%)	21 (34.4%)	12 (19.7%)	61 (100%)

### Performance of MRI in BI-RADS 4 mammographic microcalcifications

**Fig 1:**
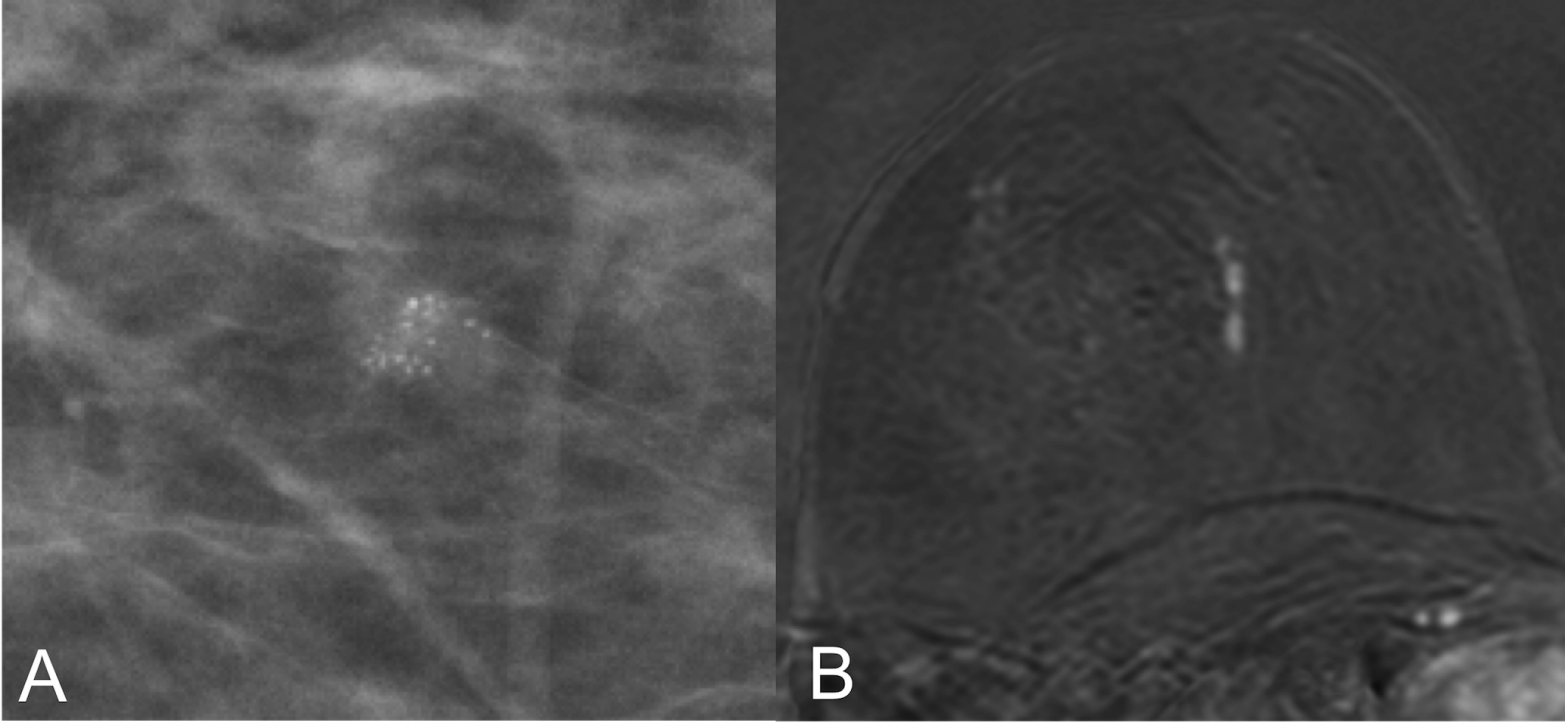
Small cluster of screening detected BI-RADS 4b microcalcifications (A) in the right breast of a 50-year old woman. MRI (B) demonstrates a linear clumped non-mass enhancement rated BI-RADS 4. Vacuum-assisted breast biopsy and subsequent surgery revealed a hormonal receptor positive DCIS G2.

**Fig 2:**
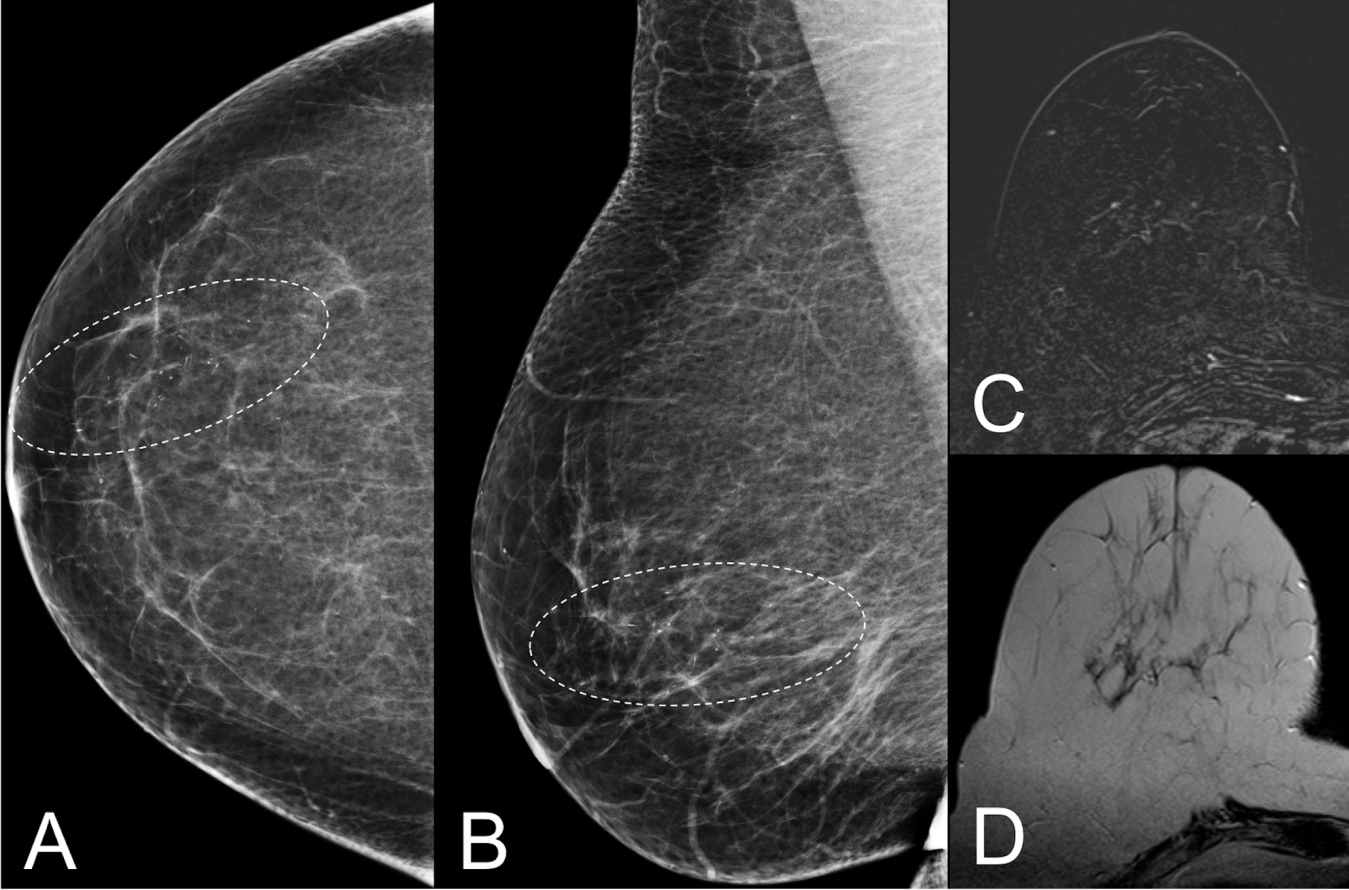
Screening detected segmental linear and coarse heterogeneous microcalcifications BI-RADS 4a in the right breast of a 59-year old woman (A, B). Contrast enhanced MRI (C) revealed no enhancing lesions, small foci were not associated with the microcalcifications. T2-weighted images did not reveal any architectural distortions (D). Histopathology revealed secretory changes, B2.

There were 103 true-positive, 116 true-negative, 25 false-positive, and 4 false-negative breast MRI findings, effecting a sensitivity, specificity, PPV, and NPV of 96.3% (95%-CI 90.7–99.0%), 82.3% (95%-CI 75.0–88.2%), 80.5% (95%-CI 72.5–87.0%) and 96.7% (95%-CI 91.7–99.1%), respectively. Among the 4 false-negative findings were one invasive cancer (G3) and three DCIS (2 G2, 1 G3). The invasive carcinoma was classified BI-RADS 4c and the 3 false negative DCIS were originally rated BI-RADS 4a (n = 1), BI-RADS 4b (n = 1) and BI-RADS 4c (n = 1) at mammography. MRI sensitivity, specificity, PPV, and NPV for invasive carcinomas were 98.8% (95%-CI 93.5–100%), 72.6% (95%-CI 65.1–69.2%), 64.8% (95%-CI 55.9–73.1%) and 99.2% (95%-CI 95.4–100%), respectively. Examples are given in Fig 1 and Fig 2.

### Performance of MRI stratified according to mammographic BI-RADS rating (4a-4c)

126 microcalcified lesions were rated BI-RADS 4a at mammography. 103 of which were benign, and 23 of which were malignant, resulting in a disease prevalence of 18.3% (95%-CI 11.9–26.1%). Calculated sensitivity, specificity, PPV, and NPV were 95.7% (95%-CI 78.1–100%), 85.4% (95%-CI 77.1–91.6%), 59.5% (95%-CI 42.1–75.3%) and 98.9% (95%-CI 93.9% to 100%), respectively.

60 lesions were classified BI-RADS 4b at mammography and harboured a disease prevalence of 41,7% (95%-CI 29.1–55.1%; 25 malignant, 35 benign findings). Resulting sensitivity, specificity, PPV, and NPV were 96.0% (95%-CI 79.7%-99.9%), 71.4% (95%-CI 53.7–85.4%), 70.6% (95%-CI 52.5–84.9%) and 96.1% (95%-CI 80.4–99.9%), respectively.

62 lesions rated BI-RADS 4c at mammography displayed a malignancy rate of 95.2% (95%-CI 86.5–99.0%, 59 malignant and 3 benign lesions). This effected a sensitivity, specificity, PPV, and NPV of 96.6% (95%-CI 88.3–99.6%), 100% (95%-CI 29.2–100%), 100% (93.7%-100%) and 60% (95%-CI 14.7–94,7%), respectively.

Chi-square test found no significant differences for sensitivity, specificity, PPV and NPV between BI-RADS 4a-4c subgroups (p>0.01).

## Discussion

Microcalcifications are a common finding at mammography and in the majority of DCIS lesions the only sign of malignancy [1–3]. While MRI features the highest sensitivity and specificity of all imaging modalities with regard to breast cancer, its potential role in the assessment of mammographic microcalcifications is still controversially discussed. One major reason undoubtedly being that calcifications themselves are not visible on MRI. Still, due to its ability to detect neoangiogenesis and the fact that lesions as small as 2-3mm are reportedly visible on MRI, the corresponding area of microcalcifications should technically be assessable by MRI [11, 14–18].

A recent meta-analysis suggested a role for MRI in the evaluation of mammographic microcalcifications rated BI-RADS 4 [5]. However, the number of available studies this finding was based on was limited (n = 4) and the cohort sizes were small, with the numbers of lesions ranging from 27–78 [9, 19–21]. The data presented herein is derived from 248 lesions and therefore the largest cohort reported on in this context so far. Previous studies reported disease prevalences ranging from 32.1% to 62.5% for BI-RADS 4 mammographic microcalcifications. Our cohort exhibits a disease prevalence of 43.1% which is well within this range. We also find a clear increase of malignancy rates from BI-RADS 4a to BI-RADS 4c lesions, with the disease prevalence being above 95% in BI-RADS 4c lesions. This finding alone indicates, that it is not useful to perform an MRI for BI-RADS 4c lesions in order to rule out malignancy as mammography is accurate enough to predict malignancy in these lesions and warrant definite management by percutaneous biopsy.

Diagnostic performance of previous studies is listed in Table 3. Our study shows comparable results regarding sensitivity and specificity. PPV and NPV are dependent on disease prevalence, and therefore always need to be evaluated in its context. Given the fact that NPV is inversely correlated with prevalence and the fact that our cohort already features a relatively high disease prevalence of 43.1%, we believe this value to reflect the true performance of MRI in this setting and if anything, to be a conservative estimate. Consequently, in our cohort MRI could exclude malignancy of BI-RADS 4 microcalcifications with a certainty of 97%. 3 of the lesions that were wrongly classified as benign on MRI were DCIS and one constituted an invasive carcinoma. Given the ongoing controversy around DCIS not always representing clinically relevant disease that will progress into cancer [22] accompanied with growing concern that many of these lesions are being overtreated/-diagnosed [23], MRI test performance for diagnosis of invasive lesions is clinically relevant. Here, MRI provides an edge over other imaging modalities since it indicates neovascularization. Neoangeogenesis has already been postulated more than 20 years ago to mark the difference between biologically active and dormant cancer [24]. Sensitivity and NPV for invasive cancer were 98.8% and 99.2% in our study. These findings confirm the results of the meta-analysis cited above [5]. The one remaining invasive lesion that went undetected by MRI was a BI-RADS 4c lesion, a lesion that given the likelihood of malignancy at mammography, would have not been missed in the diagnostic workup. Although the pooled results from all BI-RADS 4 lesions combined in the meta-analysis referenced above already suggest a role for MRI in the workup of BI-RADS 4 mammographic microcalcifications we further looked at test performance in BI-RADS 4 subgroups. Sensitivity, being independent from cancer prevalence, did not differ significantly between BI-RADS 4 subgroups, which indicates applicability of MRI in all subcategories. Specificity was above 70% in the subgroups of BI-RADS 4a and 4b mammographic microcalcifications where our results indicate the highest usefulness of additional breast MRI. Consequently, the potential value of MRI to downgrade mammographic microcalcifications (e.g. avoid biopsies in benign microcalcifications) can be considered higher than its negative impact as measured by false positive findings. In our study, all patients referred to MRI already underwent stereotactical biopsy according to the current guidelines. Thus, using MRI for further patient management could potentially have avoided 82% unnecessary biopsies in BI-RADS 4a and 58% unnecessary biopsies in BI-RADS 4b lesions. Due to the retrospective character of our study, we cannot estimate to what extent false positive findings by MRI were associated with the mammographic microcalcifications or incidental additional lesions detected by MRI. In addition, our patient-wise analysis does not cover MRI-detected additional e.g. multicentric cancer lesions or whether it caused unnecessary biopsies of additional false positive findings. A deeper analysis of the possible implications of patient-wise vs lesion-wise analysis is given in [25]. Whether the use of MRI can improve preoperative planning of malignant lesions by more accurate mapping of lesion extent remains a matter of debate and was not tested for in this study. Recent data suggest that presence of enhancement as sole diagnostic criterion may constitute the best approach to diagnosing mammographic microcalcifications at MRI [5]. Our work documents routine reading results in clinical practice and thus did not document specific diagnostic criteria that were decisive for the MRI diagnosis. This however, can be seen as a strength of this study as the setting reflects and its results apply to clinical practice while a retrospective reading setup may less do so. Also, and as it is the case for previously published studies on this topic, our results lack additional subgroup data regarding the MRI correlate of mammographic microcalcifications as e.g. mass or non-mass. It is presumable that the “presence of enhancement” criterion is more useful in the diagnostically problematic non-mass enhancements as MRI BI-RADS criteria do quite accurately allow to distinguish benign from malignant mass lesions [26–28]. Whether modified MRI protocols including first pass high temporal resolution [29, 30], functional techniques [31–33] and higher field strength [34, 35] would impact MRI performance in this setting remains to be seen in further studies. Finally, the cost effectiveness of using MRI as an additional test in mammographic microcalcifications should be critically evaluated. Given the very good diagnostic performance based on our data, this warrants more research into this direction to further refine which patients may benefit from additional MRI and which will not.

**Table 3:** Comparison of MRI performance with published data.

First author	year	lesions	prevalence	sensitivity	specificity	PPV	NPV
**Uematsu T [19]**	2007	27	0.48 (0.29–0.68)	0.92 (0.64–1)	0.79 (0.49–0.95)	0.80 (0.52–0.96)	0.92 (0.62–1)
**Jiang Y [9]**	2014	69	0.65 (0.53–0.76)	0.96 (0.85–1)	0.79 (0.58–0.93)	0.90 (0.77–0.97)	0.91 (0.70–0.99)
**Li E [21]**	2014	51	0.45 (0.31–0.60)	0.91 (0.72–0.99)	0.82 (0.63–0.94)	0.81 (0.61–0.93)	0.92 (0.74–0.99)
**Strobel K [20]**	2015	78	0.32 (0.22–0.44)	0.88 (0.69–0.97)	0.85 (0.72–0.93)	0.73 (0.54–0.88)	0.94 (0.83–0.99)
**this study**	2016	248	0.43 (0.37–0.50)	0.96 (0.91–0.99)	0.82 (0.75–0.88)	0.81 (0.73–0.87)	0.97 (0.92–0.99)

PPV: Positive Predictive Value; NPV: Negative Predictive Value

## Conclusion

MRI is an accurate tool to further diagnose BI-RADS 4a and 4b microcalcifications and may be helpful to avoid unnecessary biopsies in BI-RADS 4a and 4b lesions. BI-RADS 4c lesions should be biopsied irrespective of MRI findings.

## Supporting information

S1 File SPSS table of the raw study data. (SAV)

## Abbreviations

EIC: Invasive Carcinoma with Extensive Intraductal Component
DCIS: Ductal Carcinoma in situ
IC: invasive carcinoma
ER: Estrogen Receptor
PR: Progesterone Receptor
Her2/neu: Human epidermal growth factor receptor 2
PPV: Positive Predictive Value
NPV: Negative Predictive Value
